# In Vitro and In Vivo Dendritic Cell Immune Stimulation Effect of Low Molecular Weight Fucoidan from New Zealand *Undaria pinnatifida*

**DOI:** 10.3390/md20030197

**Published:** 2022-03-08

**Authors:** Litong Liu, Xu Yang, Pengfei Yuan, Shanshan Cai, Jing Bao, Yanan Zhao, Alimu Aimaier, Adila Aipire, Jun Lu, Jinyao Li

**Affiliations:** 1 Xinjiang Key Laboratory of Biological Resources and Genetic Engineering, College of Life Science and Technology, Xinjiang University, Urumqi 830046, China; lltong95@163.com (L.L.); 18690282867m0@sina.cn (P.Y.); 18699927521@163.com (S.C.); bj_1695354622@163.com (J.B.); zhaoyanan514@163.com (Y.Z.); alimu15276554161@163.com (A.A.); kaskas999@163.com (A.A.); 2 School of Science, Faculty of Health and Environmental Sciences, Auckland University of Technology, Auckland 1010, New Zealand; cindy.yang@aut.ac.nz; 3 School of Public Health and Interdisciplinary Studies, Faculty of Health and Environmental Sciences, Auckland University of Technology, Auckland 0627, New Zealand; 4 Maurice Wilkins Centre for Molecular Biodiscovery, Auckland 1010, New Zealand

**Keywords:** low molecular weight fucoidan (LMWF), dendritic cells, immune stimulation, *Undaria pinnatifida*, New Zealand

## Abstract

Low molecular weight fucoidan (LMWF) has been reported to have immunomodulation effects through the increase of the activation and function of macrophages. In this study, the regulating effect of LMWF from *Undaria pinnatifida* grown in New Zealand on dendritic cells (DCs) was investigated. We discovered that LMWF could stimulate DCs’ maturation and migration, as well as CD4+ and CD8+ T cells’ proliferation in vitro. We proved that this immune promoting activity is activated through TLR4 and its downstream MAPK and NF–κB signaling pathways. Further in vivo (mouse model) investigation showed that LMWF has a strong immunological boosting effect, such as facilitating the proliferation of immune cells and increasing the index of immune organs. These findings suggest that LMWF has a positive immunomodulatory effect and is a promising candidate to supplement cancer immunotherapy.

## 1. Introduction

As a natural sulfated polysaccharide, fucoidan mainly exists in the cell wall of brown algae and has a variety of biological activities, including anti-inflammatory [1], antiviral [2], antioxidant [3], anticoagulant [4], immunomodulatory [5], and antitumor activities [6]. The function of fucoidan is regulated by many conditions, including the source, harvest time, molecular weight, monosaccharide composition, degree of substitution of sulfuric acid groups, and spatial structure [7,8,9,10]. According to several previous studies, fucoidan promotes the maturation of DC by enhancing the expression of DC surface molecules and the secretion of cytokines [11,12] to further improve the intensity of immune response [13].

Dendritic cells (DCs), a link between innate immunity and adaptive immunity, play a connecting role in the occurrence and development of immune response. They are the only antigen presenting cells (APC) that can directly activate naïve T cells. Immature DCs which express high levels of pattern recognition receptors have remarkable capability to recognize and phagocytize antigens, and their phagocytic ability decreases along with the maturation of DC [14]. Meanwhile, the most important functions of DCs are antigen presentation and immune activation. However, due to the lack of maturity, DC-based vaccine is limited in clinical application. Therefore, it is necessary to develop safe, non-toxic, and efficient adjuvants to promote the maturation of DC [15]. Polysaccharides from natural sources have been extensively studied because of their wide range of biological activities and low toxicity [16].

Therefore, this paper investigated the regulation of a fraction of marine natural polysaccharide–low molecular weight fucoidan (LMWF) on DCs and the promoting effect of fucoidan on the maturation and function of DC in vivo and in vitro, which will lay the groundwork for further development and utilization of LMWF.

## 2. Materials and Methods

### 2.1. Materials

The LMWF was obtained from New Zealand *Undaria pinnatifida* as described previously [17]. RPMI-1640 medium and phosphate-buffered solution (PBS) were bought from Gibco (Gaithersburg, MD, USA). Lipopolysaccharides (LPS), polymyxin B (PMB), and FITC-DEXTRAN (42,000 Da) were purchased from Sigma-Aldrich (St. Louis, MO, USA). Granulocyte-macrophage colony-stimulating factor (GM-CSF) and TAK-242 were purchased from PeproTech (Rocky Hill, NJ, USA) and Medchemexpress (Monmouth Junction, NJ, USA), respectively. 

### 2.2. Animals and Ethics

ICR and C57BL/6 mice (6–8 weeks) were bought from Animal Laboratory Center, Xinjiang Medical University (Urumqi, Xinjiang, China) and housed in a standard temperature-controlled, light-cycled animal facility at Xinjiang University. The animal experiment (BRGE-AE001-075) was approved by the Committee on the Ethics of Animal Experiments of Xinjiang Key Laboratory of Biological Resources and Genetic Engineering (BRGE-AE001) and carried out under the guidelines of the Animal Care and Use Committee of the College of Life Science and Technology, Xinjiang University.

### 2.3. DC Treatment 

Immature DCs (iDCs) were obtained from bone marrow cells of C57BL/6 mice according to our previous elaboration [18]. Briefly, the femurs and tibias of mice were collected and put into 75% ethanol solution for 3 min. After washing with PBS 3 times, bone marrow cells were taken with a syringe, then a single cell suspension was prepared. After centrifuging at 1200 rpm/min for 7 min, the supernatant was removed and cells were suspended in 6 mL of RPMI-1640 medium containing 10% fetal bovine serum, 1% penicillin-streptomycin, and 20 ng/mL GM-CSF, then it was cultured at 37 °C in an incubator with a 5% CO_2_ atmosphere. Then, 3 mL of medium was removed and the fresh culture medium was added the next day. On the third day, the supernatant was removed and the complete 6 mL of medium was supplemented, then, the medium was half-changed again on the fifth day. On day 7, iDCs were collected and treated with different concentrations (20, 50, and 100 μg/mL) of LMWF for 12 h, and LPS (40 ng/mL) was used as the positive control. For the detection of phagocytosis ability, iDCs were treated with different concentrations (20, 50, and 100 μg/mL) of polysaccharides for 12 h and then co-incubated with FITC-DEXTRAN for 1 h at 37 °C. The cells were then collected and treated with precooling PBS to terminate the reaction. In endotoxin assay, DCs were pretreated with or without PMB (10 μg/mL) for 2 h, and then treated with LPS or LMWF (100 µg/mL) for 12 h. PMB was adopted as an endotoxin inhibitor in this paper to exclude the influence of endotoxin on the maturation of DC [19]. DCs were pretreated with or without 1 μM TAK-242 for 1 h and the supernatant was removed, then, the cells were re-suspended in fresh medium without TAK-242, followed by the treatment of LMWF (20, 50, and 100 μg/mL) for 12 h for TLR4 inhibition treatment. 

### 2.4. Enzyme-Linked Immunosorbent Assay (ELISA)

After treatment by LPS and LMWF (20, 50, and 100 μg/mL), we collected the supernatant of DCs and detected the level of interleukin (IL)-12p40 and tumor necrosis factor-α (TNF-α) via an ELISA kit following the manufacturer’s protocol (Elabscience, Houston, TX, USA), Briefly, the supernatants of LPS and LMWF treated DCs (10^6^ cells/mL) were diluted 10 times. After incubating at 37 °C for 90 min, supernatants were removed and 100 μL of antibody solution was added. After incubation for 1 h at 37 °C, 250 μL of washing buffer was added to each well. Soaked for 1 min and the solution was decanted from each well and patted dry on absorbent paper. After washing three times, 100 μL of enzyme binding working solution was added and incubated for 30 min at 37 °C, followed by the above washing steps for five times. Then, 90 μL of substrate reagent was added, and incubated for 15–20 min at 37 °C. A volume of 50 μL of stop solution was added to terminate the reaction and the absorbance value was detected at 450 nm. The levels of IL-6 in serum were also detected by ELISA kit according to the above procedure. ELISA kits for murine TNF-α, IL-12p40, and IL-6 were purchased from Elabscience (Wuhan, China).

### 2.5. Mixed Lymphatic Reaction (MLR)

The induced DCs were collected from C57BL/6 mice on day 7, treated with LPS or different concentrations of LMWF for 12 h. The freshly ground spleen homogenate from ICR mice was stained with carboxyfluorescein diacetate succinimidyl ester (CFSE) (eBioscience), and then the splenocytes and DCs were co-cultured at the ratio of 1:5 in a 37 °C incubator with a 5% CO_2_ atmosphere for 72 h.

### 2.6. Animal Model

For the experiment of DC maturation in vivo, 12 ICR mice of about 20 g were randomly divided into three groups, including untreated, treatment of LPS (100 ng/mouse, as the positive control), and LMWF (500 μg/mouse). The mice were injected by footpad inoculation and sacrificed by cervical dislocation 24 h later. The draining lymph nodes were taken, grounded, washed with PBS, and stained for flow cytometry analysis. For in vivo migration experiment, iDCs were treated with or without LPS (40 ng/mL) or LMWF (50 μg/mL) for 12 h and then stained with 1 μM CFSE for 10 min. After washing with PBS, cells were re-suspended in PBS at 10^6^ cells/100 μL PBS. Untreated iDCs labeled with CFSE was named as iDCs. Unlabeled iDCs were used as blank control and named as untreated. The in vivo migration experiment was conducted by intraperitoneal injection. After injection of unlabeled iDCs and CFSE-labeled DCs, the inguinal lymph nodes of mice were collected and analyzed. 

Twenty ICR mice with a body weight of about 20 g were randomly divided into five groups, including untreated, λ-carrageenan (λ-CGN, 20 mg/kg), a low dose of LMWF (20 mg/kg), a medium dose of LMWF (40 mg/kg), and a high dose of LMWF (80 mg/kg). λ-CGN was used as the positive control as described previously [20]. Mice in untreated group did not receive additional intervention. Mice were injected intraperitoneally at day 0, 2, 4, 6, and 8. On day 10, all mice were sacrificed by cervical dislocation and organs were collected and weighted. Splenocytes were used to detect the proportions of immune cells by flow cytometry.

Cyclophosphamide (CTX) is a widely used antineoplastic drug and immunosuppressant in clinic [21,22]. Long-term use of cyclophosphamide will bring serious side effects, including myelosuppression, immunosuppression, and leukopenia [23]. It is often used to induce immunosuppressive models. We injected mice intraperitoneally with 80 mg/kg of CTX for four consecutive days to establish an immunosuppressive model. The untreated group did not receive additional intervention, the CTX group was injected by CTX without drug treatment, while the other treatment groups were treated with drug after injection of CTX. Mice showed obvious weight loss and mental malaise. On the next day after CTX injection, mice in treatment groups received intraperitoneal injection of λ- CGN or LMWF every other day. When the body weight of the treatment group recovered similar to that of the untreated group, the experiment was terminated. Organ index = visceral mass (mg)/body weight (g).

### 2.7. Western Blot

After treated with 50 μg/mL of LMWF, DCs were collected at different time points (0, 30, 60, 120, and 240 min) to extract proteins using the Nuclear and Cytoplasmic Protein Extraction Kit (Beijing TransGen Biotech). Then, the protein concentrations were analyzed by the BCA Kit (Thermo Fisher Scientific, Waltham, MA, USA) according to the manufacturer’s instructions. Equal amounts of protein from cell extracts were loaded and electrophoresed on a gel (SDS-PAGE). After SDS-PAGE, proteins were transferred to PVDF membrane, and then blocked in 5% skimmed milk powder in a 37 °C incubator for an hour. After blocking, PBST (PBS-containing 0.05% Tween-20) was used to wash the membrane three times, 15 min for each wash. Then, the membrane was incubated with anti-β-actin (1:1000), JNK (1:1000), p-JNK (1:1000), p38 (1:1000), p-p38 (1:1000), NF–κB p65 (1:500), p-NF–κB p65 (1:500), iκi (1:500), p-iκ1 (1:500) ERK (1:1000), or p-ERK (1:1000) (Cell Signaling Technology, Danvers, MA, USA) at 4 °C overnight, washed three times, and incubated for an additional hour with corresponding peroxidase-conjugated HRP (1:1000) labeled secondary antibodies (Beyotime Biotech Co., Ltd. Shanghai, China) at 37 °C. After that, the membrane was washed three times. Finally, the membrane was viewed on Las 4000 (FVJIFILM corporation, Tokyo, Japan).

### 2.8. Flow Cytometry

After various treatments, the DCs were washed with PBS, and then the DCs were stained with various antibodies labeled with different fluorescence according to previously published method [24]. For the expression of co-stimulatory, MHC molecules, and CCR7, the fluorescence-conjugated antibodies used were APC-CD40 and PerCP-CD86 or FITC-MHCⅠ and APC-MHC-Ⅱ (all from Elabscience Wuhan, Hubei Province, China) or PE-CCR7 (BD Biosciences, San Diego, CA, USA). We used PE-CD4 and APC-CD8 (Elabscience Wuhan) to detect the ratio of T cell proliferation. For the proliferation of immune cells in mouse spleen, we used FITC-CD49b and FITC-CD11c (BD Biosciences, San Diego, CA, USA), and APC-CD3, PE-CD19, APC-CD86, PE-CD11b, APC-CD8, FITC-CD4, and PE-CD44 (Elabscience Wuhan) stained splenocytes. The relevant indexes in the immunosuppression model were detected by APC-CD19, PerCP-CD3, FerCP-CD11c, FITC-CD11b, APC-CD8 and PE-CD4 (Elabscience Wuhan), and FITC-CD49b (BD Biosciences). A volume of 0.25 μL of antibody per sample in vitro and 0.5 μL of antibody per sample in vivo were used. All samples were collected on FACSCalibur (BD Biosciences) and the data were analyzed by the FlowJo platform (Tree Star, Inc., Ashland, OR, USA). 

### 2.9. Statistical Analysis

The data were expressed as mean ± SD. Statistical analysis was carried out by one-way analysis of variance (ANOVA) or unpaired *t*-test using Prism8.0 software. *p* < 0.05 was considered statistically significant.

## 3. Results and Discussions

### 3.1. LMWF Can Stimulate the Maturation of DCs In Vitro and In Vivo

**Figure 1:**
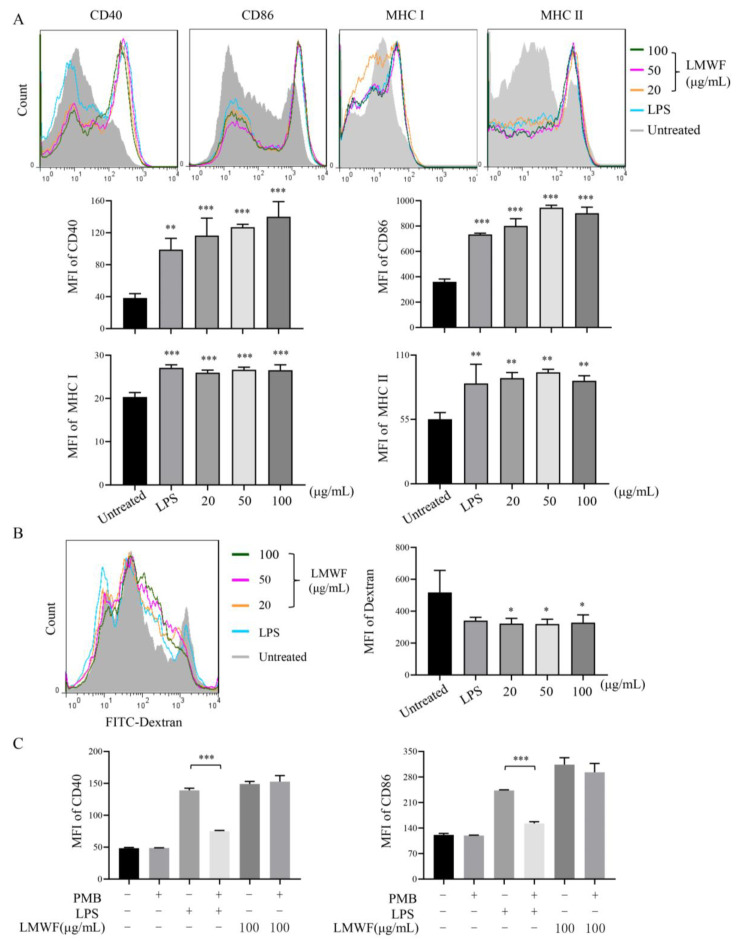
LMWF’s ability to promote DC maturity in vitro. After 12 h of treatment with different concentrations of LMWF, flow cytometry was used to detect the expression of surface molecules in DC. LPS (40 ng/mL) was used as the positive control. (**A**) Expression of CD40, CD86, MHCI, and MHCII (upper part) and mean fluorescence intensity (MFI) (lower part). (**B**) LMWF reduced the phagocytosis of DC, after treatment, FITC labeled Dextran was added to detect the phagocytosis efficiency of DC. (**C**) Endotoxin Pollution Assay of LMWF. PMB can neutralize endotoxin by acting on the cell wall of gram-negative bacteria, so we used PMB to detect the presence of endotoxin contamination in LMWF. Data are from 3 independent experiments and analyzed by prism8.0 software. * *p* < 0.05; ** *p* < 0.01; *** *p* < 0.001 compared to untreated DCs.

**Figure 2:**
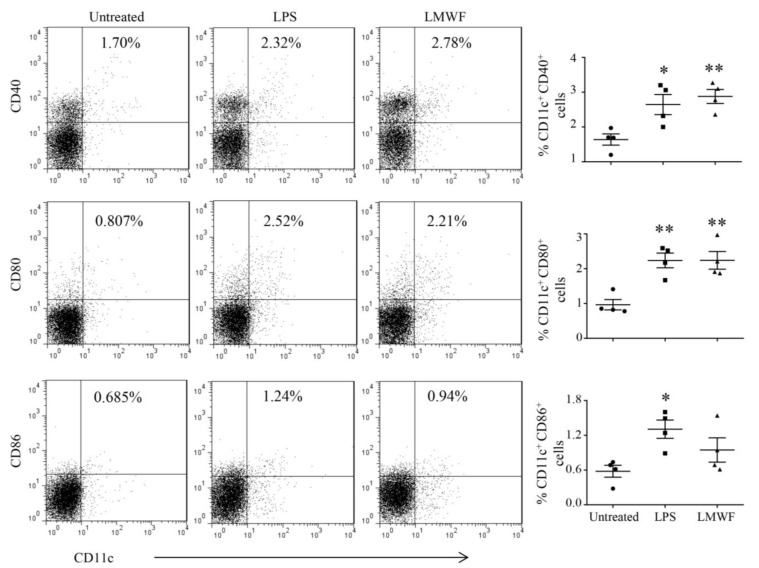
LMWF promotes DC maturation in vivo. Mice were injected with 500 μg of LMWF by footpad inoculation, and 100 ng of LPS was used as a positive control. After 24 h, draining lymph nodes were taken to detect the surface molecules that marked the maturity of DC by flow cytometry. * *p* < 0.05; ** *p* < 0.01 compared to untreated.

To evaluate the effect of LMWF on DC maturation in vitro, DCs induced to the seventh day were treated with different concentrations of LMWF for 12 h, LPS (40 ng/mL) was used as the positive control, and the expression of CD40, CD86, and MHC molecules were detected by flow cytometry. The results showed that LMWF significantly enhanced the expression of CD40, CD86, MHC Ⅰ, and MHC Ⅱ (Figure 1A). DCs have strong antigen phagocytosis ability at the immature state, but with the maturity of DCs, this ability will be weakened. Therefore, this can be used as an indication of DC maturity. As shown in Figure 1B, when compared to control, LMWF could significantly reduce the phagocytic ability of DC. Results indicated that LMWF could stimulate the maturation of DCs in vitro. In the meantime, we also examined the effect of LMWF on DC maturation in mice. We found that LMWF could also promote the maturation of DC in vivo, significantly increased the expression of CD40 and CD80 compared to untreated mice (Figure 2). Although the expression of CD86 in mice was not statistically significant, its level also showed an increasing trend (Figure 2).

This finding was also reported in a previous study [12], human monocyte-derived immature DCs were grown with fucoidan to study the direct influence of fucoidan on the maturation of sentinel DCs into effector DCs. In contrast to unstimulated cells, fucoidan treatment promoted the expression of CD83 and increased the expression of CD80, CD86, and HLA-DR within 48 h, in the same way that LPS treatment did. These findings indicated that fucoidan has the ability to cause immature DCs to develop [12]. However, this study did not perform the endotoxin control experiment. Our study, on the other hand, used an endotoxin-free LMWF. HMWF may contain endotoxin, which could lead to false positive results.

In order to rule out the false positive results caused by endotoxin; we performed an endotoxin test that the endotoxin antagonist, PMB, was used to pretreat DCs (Figure 1C). The result showed that the function of LPS was significantly inhibited after PMB treatment, but there was no change in LMWF induced increase with the presence of PMB. Therefore, it is indicated that LMWF used in this experiment does not contain endotoxin.

### 3.2. LMWF Enhances the Migration of DCs In Vitro and In Vivo

**Figure 3:**
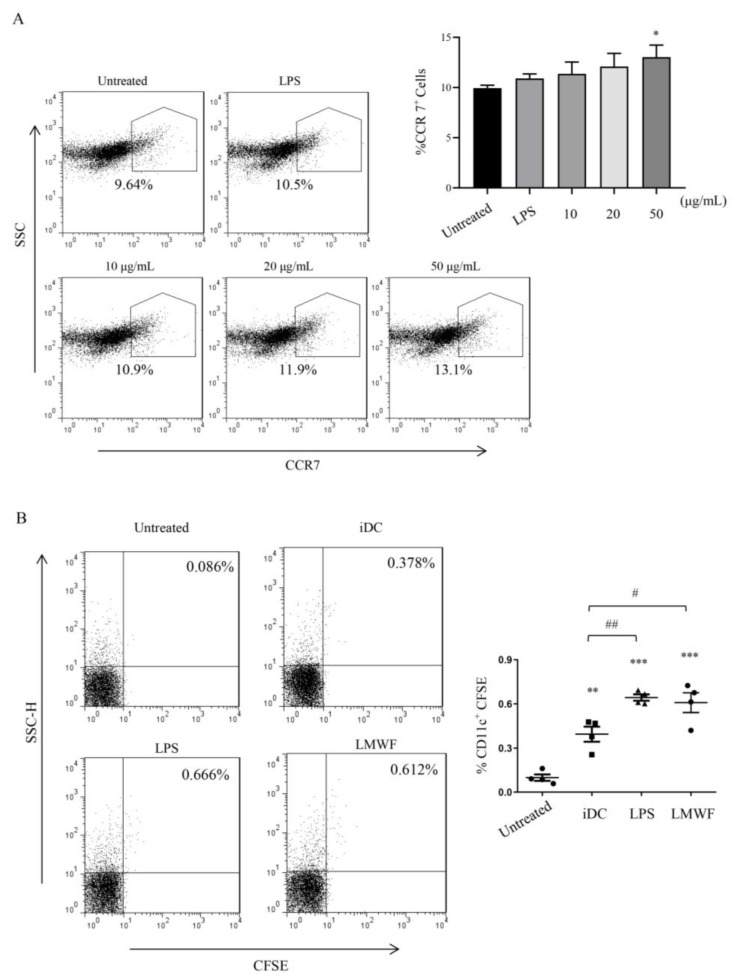
LMWF promotes DC migration in vitro and in vivo. (**A**) After being treated with different concentrations of LMWF, the expression of CCR7 was detected by flow cytometry, and the percentage of CCR7 cells was counted. (**B**) DC was labeled with CFSE. After LMWF treatment, the cells were collected and conducted by intraperitoneal injection. The proportion of CD11c+ CFSE cells in inguinal lymph nodes was calculated, LPS was used as positive treatment. DC labeled only with CFSE was iDC group, and unlabeled iDC was untreated group as blank. * *p* < 0.05; ** *p* < 0.01; *** *p* < 0.001 compared to untreated DCs, # *p* < 0.05; ## *p* < 0.01 compared to iDC.

DCs are migratory immune cells with phagocytic and antigen-presenting capabilities. The migration ability of DC essentially contributes to initiate immune response and exercise immune surveillance function [25]. Thus, we planned to detect the expression of DC surface receptor CCR7 after treated with LMWF for 12 h. We found that LMWF (50 μg/mL) could significantly enhance the expression of CCR7 in vitro (Figure 3A), and the experiment in vivo showed similar results, LMWF could significantly increase the expression of CFSE-conjugated CD11c+ (Figure 3B). The results suggest that LMWF enhances the migration of DCs.

In contrast, Park et al. reported the experiment with different molecular weight fucoidan from *U. pinnatifida* [26]. HMWF (100 ± 4 kDa) up-regulated the intercellular adhesion molecule (ICAM)-1 and CD11a expression levels to a similar extent as that of LPS, according to this study. Treatment with either medium molecular weight fucoidan (MMWF, 3.5 ± 0.3 kDa) or LMWF (1 ± 0.2 kDa) resulted in just a minor rise in ICAM-1 expression levels and no increase, even a slight drop, in CD11a expression levels [26]. A possible reason for this may be due to the trace existence of LPS in HMWF triggers immunological response. Therefore, a further in-depth investigation of HMWF is required. As we have performed the LPS contamination control, our results should be credible.

### 3.3. LMWF Promotes DC Maturation via TLR4 Signaling Pathway

**Figure 4:**
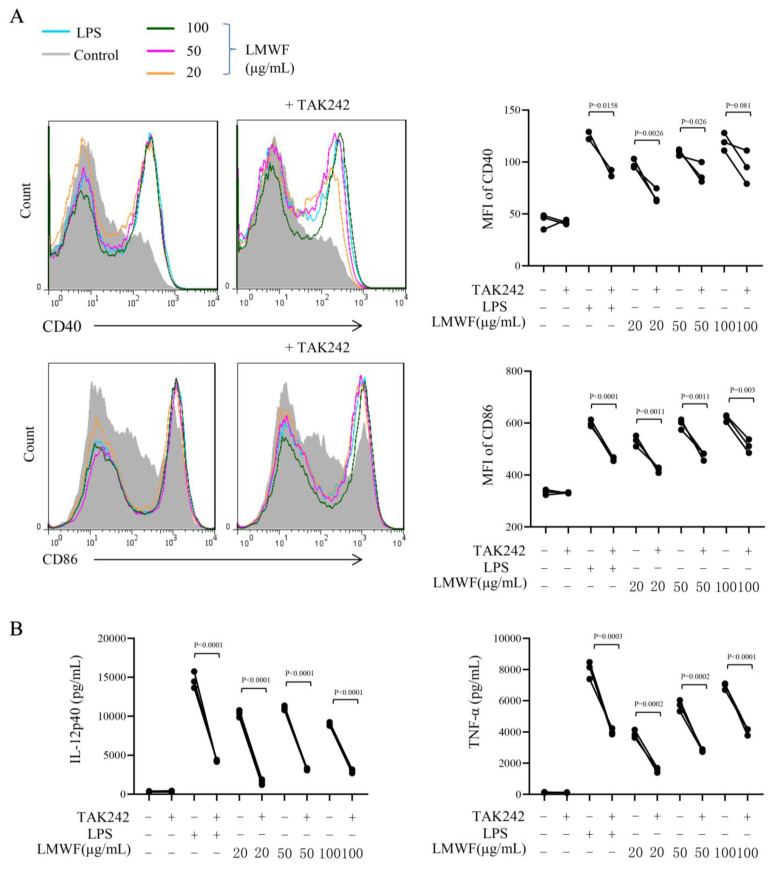
LMWF stimulate the maturation of DC via TLR4 signaling pathway. DC was pretreated by the inhibitor of TLR4 (TAK-242), followed by the treatment of LMWF for 12 h. (**A**) The expression of surface molecules were detected by flow cytometry and (**B**) the secretion of IL-12p40 and TNF-α in the supernatant were detected by ELISA. The data are from 3 independent experiments and analyzed by prism8.0 software.

TLR4 has been widely studied in tumor immunotherapy, especially in the immune response induced by polysaccharides [27,28]. Our prior research has validated this conclusion [29]. We further hypothesize that LMWF also activates DC through this pathway to exert its immune-modulation effect. We used TAK-242, the inhibitor of TLR4 (1 μM), pretreated DC for 1 h, followed by the treatment of LMWF for 12 h and then collected DCs. The expressions of surface molecules were detected by flow cytometry, and the secretion of cytokines in the supernatant were detected by ELISA. The results demonstrated that the expression of CD40 and CD86 and secretion of IL-12 and TNF-α were significantly inhibited by the addition of inhibitor (Figure 4). It showed that LMWF’s promotion of DC maturation and cytokines production is mediated by TLR4. 

**Figure 5:**
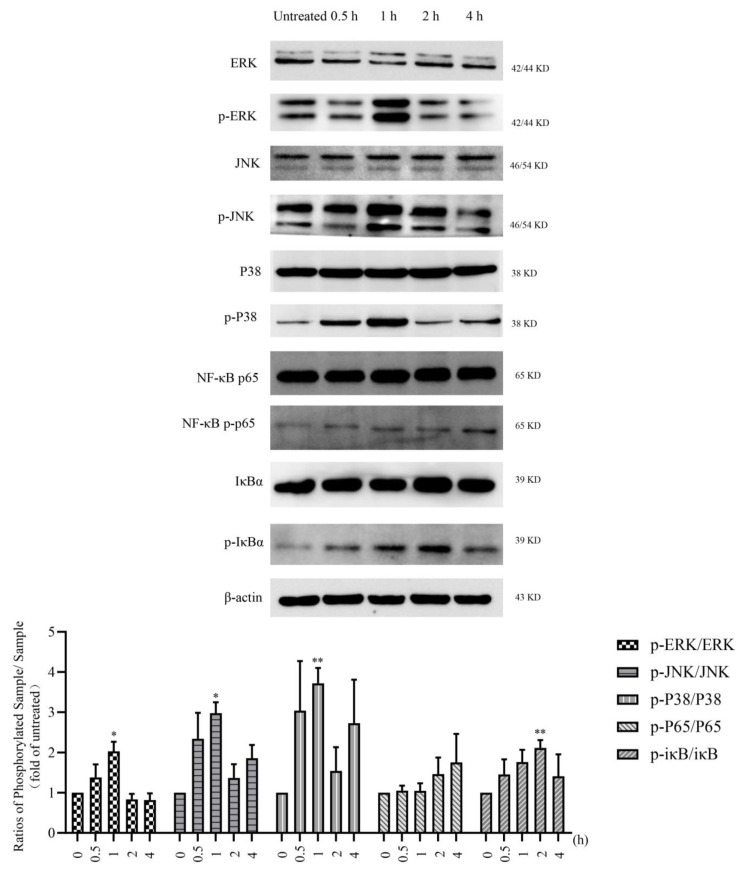
After treated with 50 μg/mL of LMWF, DCs were collected at different time points (0, 30, 60, 120, and 240 min) to extract proteins, Western blot was used to determine the expression levels of related proteins. The results were subsequently quantified by gray-scale scanning of the bands using ImageJ. * *p* < 0.05; ** *p* < 0.01 compared to untreated.

Based on these results, we further explored the MAPK and NF–κB signaling pathways downstream of TLR4. We detected the effect of LMWF treatment on the expression of proteins related to these two signaling pathways by using the Western blot method. Phosphorylation levels of ERK, JNK, and p38 were significantly increased after LMWF treatment for 0.5 and 1 h, and reached the peak at 1 h (Figure 5). In addition, the level of p-iκB increased from 0.5 to 2 h before decreasing and the level of p-NF–κB p65 increased slowly from 2 to 4 h (Figure 5). Therefore, LMWF promotes the maturation of DC and exerts the function of immune enhancement by activating and regulating downstream signal pathways via TLR4.

These results are in consistence with those of Yang et al. who also found that in contrast to unstimulated cells, fucoidan (from *Fucus vesiculosus*) treatment induced the expression of CD83 and increased CD80, CD86, and HLA-DR expression within 48 h, in a manner similar to that seen in the LPS-treated group [12]. These findings suggest that fucoidan can cause immature DCs to mature by increasing the expression levels of immune-stimulation and maturation related surface markers [12]. This is also consistent with an earlier observation, where fucoidans from *Laminaria japonica*, *Laminaria cichorioides*, and *Fucus evanescens* can activate NF–κB through TLR-2 and TLR-4 on HEK293 cells, and the TLRs have different affinity to differently originated fucoidans [30]. 

### 3.4. LMWF Shows Capability to Induce the Proliferation of Allogeneic T Cells

**Figure 6:**
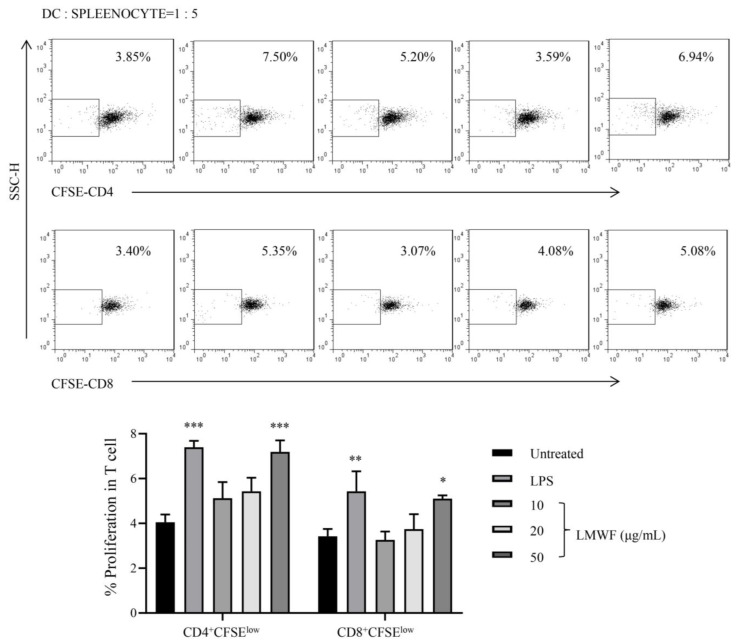
LMWF shows capacity to induce the proliferation of allogeneic T cells. MLR was performed using C57BL/6 DCs and ICR splenocytes. DCs on day 6 were treated with different concentrations (10, 20, and 50 μg/mL) of LMWF or LPS for 12 h. DCs and splenocytes were co-cultured at a ratio of 1:5 for 72 h. T cell proliferation was assessed by flow cytometry, using prism8.0 software for statistical analysis. * *p* < 0.05; ** *p* < 0.01; *** *p* < 0.001 compared to untreated.

The effect of LMWF-treated DC on the proliferation of allogeneic T cells was investigated using MLR. The CFSE-labeled splenocytes and DCs were co-cultured at the ratio of 1:5 for 72 h. The proliferation of CD4+ T cell and CD8+ T cells was detected by flow cytometry. We observed that DC treated with 50 μg/mL of LMWF can significantly enhance the proliferation of splenocytes (Figure 6).

Jin et al. found that fucoidan functions as an adjuvant to enhance ovalbumin-specific antibody production and T cell responses in vivo [31]. Fucoidan was employed as an adjuvant in vivo with ovalbumin (OVA) antigen and increased OVA-specific antibody formation as well as primed IFN-production in OVA-specific T cells [31]. Fucoidan also aided OVA-induced MHC class I and II upregulation on spleen cDCs, as well as the proliferation of OVA-specific CD4 and CD8 T cells. Finally, mice were protected from B16-OVA tumor cells after receiving OVA immunization with fucoidan as an adjuvant. These findings suggest that fucoidan can be used as an adjuvant to produce a Th1 immune response and CTL activation, which could be valuable in the development of tumor vaccines [31]. However, LMWF has not been investigated in vivo. Our study was the first study to investigate LMWF and produced similar results in comparison to Jin et al. It is worth noting that LMWF is more absorbable in human intestine which makes it a more favorable orally administered agent. In future studies, we will investigate the LMWF mechanisms of anti-tumor activity in a mouse tumor model by oral administration.

### 3.5. Immune Enhancement Effect of LMWF on Murine Immunity

**Figure 7:**
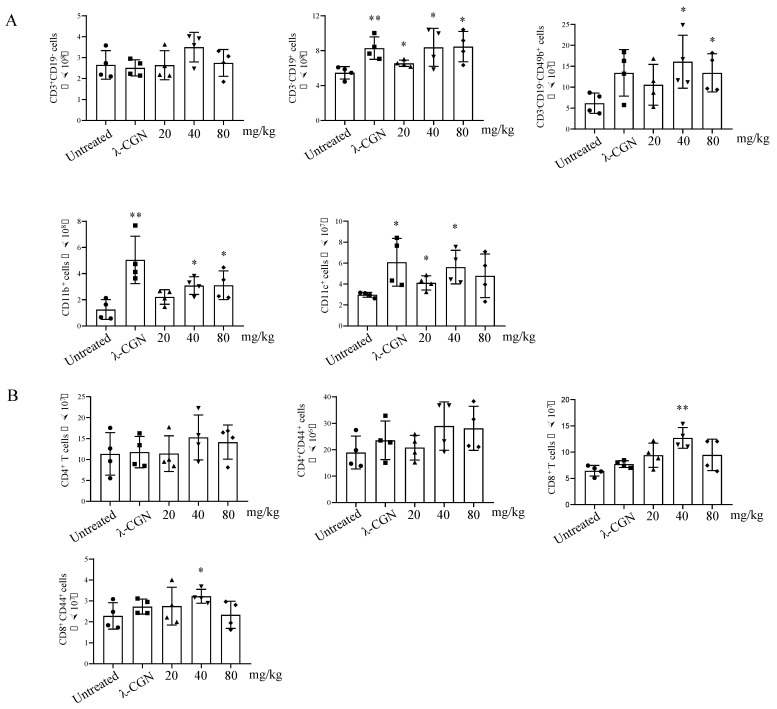
LMWF enhanced immunity in naïve mice. The mice were intraperitoneally injected with LMWF (20, 40, and 80 mg/kg) at day 0, 2, 4, 6, and 8, all mice were sacrificed, then organs were collected and weighted, and splenocytes were used to detect the proportions of immune cells by flow cytometry. (**A**) The number of T cells, B cells, NK cells, macrophages, and DCs in spleens were calculated. (**B**) The number of CD4+ T cells, CD8+ T cells and two types of cells in the activated state were calculated in spleen. * *p* < 0.05; ** *p* < 0.01 compared to untreated.

**Figure 8:**
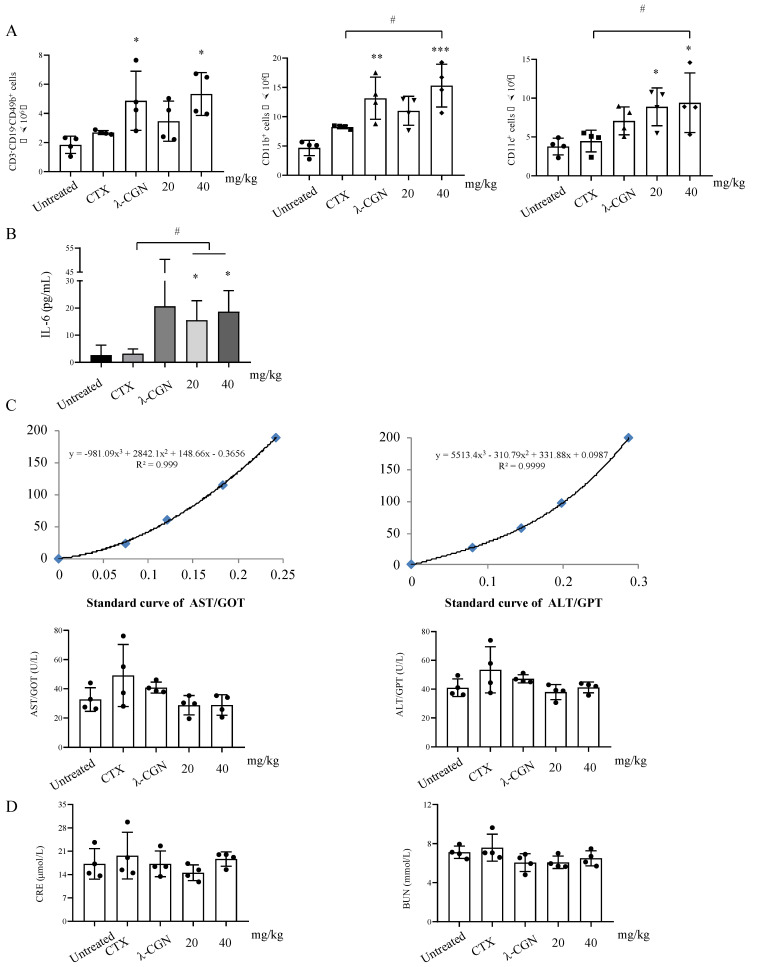
Immunoenhancement effect of LMWF on immunosuppressive mouse model. CTX was used to induce immunosuppression in mice, and LMWF was injected once every other day. At the end of the experiment, the organs and serum of mice were collected to detect the organ index of immune organs, (**A**) the proliferation of immune cells, (**B**) the production of IL-6 in serum, and (**C**,**D**) the toxicity of drugs to liver and kidney. * *p* < 0.05; ** *p* < 0.01; *** *p* < 0.001 compared to untreated. # *p* < 0.05 compared to Non-therapeutic CTX group.

The aforementioned findings show that LMWF promotes DC maturation and immune regulation in vitro. Therefore, we further verified this observation in a mouse model. Mice were injected with different doses (20, 40, and 80 mg/kg) of LMWF. We found that both low-dose and high-dose groups had significantly increased the spleen index, and there were no significant changes in the body weight (Table 1). In addition, we also examined the change of immune cells in spleen of mice. Results showed that LMWF could significantly increase the number of B cells (CD3-CD19+), NK cells (CD3-CD19-CD49b+), macrophages (CD11b+), and DC (CD11c+) in splenocytes (Figure 7A). Furthermore, CD8+ T cells, CD4+ T cells and their activation status were detected. CD8+ T cells and activated CD8+ T cells (CD8+ CD44+) were significantly increased by LMWF treatment at 40 mg/kg (Figure 7B). In order to further verify the immune enhancement effect of LMWF in mouse, CTX was used to induce immunosuppression. LMWF injection (once every other day) could significantly improve the weight loss and depilation caused by CTX. The organ index showed that the spleen was significantly enlarged after LMWF treatment (Table 2). Then the immune cells in the spleen were detected by using flow cytometry. It was found that LMWF could significantly increase the number of NK cells, macrophages and DCs, compared with that of the control group (Figure 8A). Treatment groups also had significantly higher secretion of IL-6 (Figure 8B). The evaluation of liver and kidney function showed that LMWF had no toxic or adverse effects in vivo (Figure 8C,D). Hence, we concluded that LMWF has a good immunomodulatory effect and the optimal dose of LMWF in mouse is 40 mg/kg.

However, the organ index showed that the spleen in the CTX group was significantly larger than that of the control group, which seemed to correspond to the weight gain in this group at the end of the experiment. Ding et al. [32] and Ahlmann et al. [33] pointed out that the immunosuppression caused by CTX could be restored within 10 days in mouse. Therefore, we believe that our observation is related to the slow recovery in the CTX group. The detection of liver and kidney toxicity also showed that although the indexes in CTX group improved slightly, there was no statistically significant difference (Figure 8C,D). Results of liver and kidney toxicity tests at 20 mg/kg and 40 mg/kg of LMWF treatment were almost the same as those in the control group, suggesting that LMWF might have a mitigating effect on the CTX toxicity.

Interestingly, recent studies showed that HMWF exerts a greater effect than LMWF, even though all fucoidan and its fractions increased the viability of spleen cells [34]. In addition, nuclear staining and flow cytometry analysis revealed that HMWF consistently increased spleen cell viability to a greater degree than LMWF did [34]. The reason for this could be that fucoidans extracted from different species of seaweed have different capability in immunological regulation, which is most likely owing to the difference in their chemical/structural composition [11]. Also, endotoxin may exist HMWF which may provide false positive results. Another possibility is that HMWF may interact more with gut microbials to exert its effect. While LMWF is more likely to be directly absorbed to exert its effect.

## 4. Conclusions

Despite the fact that both HMWF and LMWF have been proven to have a variety of prominent biological activities, it is important to note that the HMWF has physical and chemical shortcomings which prevent its use in medicine [35]. LMWF on the other hand has favorable physical, chemical, and biological characteristics which make it more likely to be adopted in the clinical setting. Therefore, we focused on LMWF extracted from New Zealand *U. pinnatifida*, and conducted an in-depth study on its immunomodulatory activity. Our results showed that LMWF can promote DC maturation and immune-enhancement both in vitro and in vivo, possibly through TLR4 pathway and its downstream MAPK and NF–κB signaling pathways. In general, our study demonstrates that LMWF extracted from New Zealand *U. pinnatifida* can activate the immune response through DCs. With immune enhancement activity and low toxicity, LMWF has the potential to be used as an adjuvant in cancer immune therapy and other biomedical and/or nutraceutical applications.

## Figures and Tables

**Table 1:** Organ indexes of naïve mice.

	Untreated	λ-CGN	20 mg/kg	40 mg/kg	80 mg/kg
Heart	5.03 ± 0.44	5.09 ± 0.16	5.08 ± 0.79	5.94 ± 0.37	5.64 ± 0.66
Liver	63.80 ± 3.64	72.23 ± 4.32 *	65.99 ± 5.01	71.19 ± 1.52	70.11 ± 7.24
Spleen	3.82 ± 0.84	8.76 ± 1.29 ***	6.32 ± 1.37 *	6.29 ± 0.63 *	6.81 ± 0.67 **
Lung	8.42 ± 0.67	7.00 ± 0.23	8.42 ± 1.17	8.17 ± 0.40	8.07 ± 0.90
Kidney	13.31 ± 0.64	13.28 ± 1.02	13.50 ± 0.86	14.50 ± 0.84	14.24 ± 0.85
Thymus	3.10 ± 0.53	3.29 ± 0.76	3.36 ± 0.69	5.02 ± 0.73 *	4.28 ± 1.29

* *p* < 0.05; ** *p* < 0.01; *** *p* < 0.001 compared to untreated group.

**Table 2:** Body mass index, spleen and thymus index.

	Body Weight (g)	Spleen Index (mg/g)	Thymus Index (mg/g)
Control	25.73 ± 1.24	4.36 ± 0.32	4.49 ± 0.87
CTX	18.57 ± 4.99 *	11.04 ± 0.46 **	3.88 ± 1.52
λ-CGN	20.83 ± 2.77	13.13 ± 2.60 ***	4.44 ± 1.28
20 mg/kg	22.87 ± 0.88	16.85 ± 2.59 ***^/#^	3.62 ± 0.29
40 mg/kg	20.66 ± 2.13	19.65 ± 2.34 ***^/###^	2.95 ± 1.03

* *p* < 0.05; ** *p* < 0.01; *** *p* < 0.001 compared to untreated group. ^#^ *p* < 0.05; ^###^ *p* < 0.001 compared to CTX group.

## Data Availability

Data will be made available by reasonable request to the corresponding authors.
